# “After a long day of play, I get tired and forget to unfurl my bednet”: a qualitative study exploring factors affecting insecticide-treated bednet use among school-aged children in eastern

**DOI:** 10.21203/rs.3.rs-6623966/v1

**Published:** 2025-05-13

**Authors:** Deborah Ekusai Sebatta, Sarah Alexander, John Rek, Maato Zedi, Emmanuel Arinaitwe, Mallory O. Johnson, Joaniter I. Nankabirwa, Moses Kamya, Grant Dorsey, Paul J. Krezanoski

**Affiliations:** Infectious Diseases Research Collaboration; Emory University; Infectious Diseases Research Collaboration; Infectious Diseases Research Collaboration; Infectious Diseases Research Collaboration; University of California, San Francisco; Makerere University College of Health Sciences; Makerere University College of Health Sciences; University of California, San Francisco; University of California, San Francisco

**Keywords:** malaria control, insecticide-treated bednets, qualitative research

## Abstract

**BACKGROUND:**

Despite widespread access to long-lasting insecticidal nets, infection with malaria parasites remains prevalent among African school-aged children. Though this group contributes significantly to malaria transmission, few studies have explored why their bednet use is inconsistent especially from the perspective of the children themselves. This qualitative study sought to understand the behavioral and contextual factors influencing bednet use among school-aged children in Eastern Uganda.

**METHODS:**

A cross-sectional qualitative study was conducted among children aged 8–17 years from 12 households participating in a cohort study with electronic bednet monitoring. Thirteen children (mean age 12.5 years) completed in-depth interviews, which were conducted in English, Dhupadhola, Luganda, and Ateso as appropriate. All interviews were recorded, transcribed, and translated into English. An Information-Motivation-Behavioral Skills (IMB) framework was utilized, and analysis was mapped onto its components. Themes were developed iteratively, including both inductive and deductive coding, with results presented according to the COREQ checklist. Four overarching themes were explored: barriers, facilitators, moderating factors, and proposed resolutions.

**RESULTS:**

Barriers to consistent bednet use included fatigue (e.g. after a long day of play some children forget to unfurl their bednets), parental control of nets, shared sleeping spaces, negative attitudes (e.g., discomfort, heat), and knowledge gaps. Facilitators included malaria knowledge, parental support, availability of nets, and awareness of the electronic monitoring devices. Moderating factors included age (older children showed greater autonomy), education, nightly movement, and household structure. Children proposed strategies like early net deployment, maintaining extra nets, and encouraging parents to reinforce net use routines. Notably, children perceived parents—especially mothers—as crucial in determining whether nets were unfurled and ready for use.

**CONCLUSIONS:**

School-aged children face a unique set of personal, social, and structural challenges to bednet use. Fatigue and shared sleeping spaces contribute to inconsistent usage, even when nets are available. Parental involvement is a critical determinant, especially for younger children. Public health interventions should address behavioral habit formation, intra-household net prioritization, and consistent support from caregivers to improve bednet use in this group. Engaging children directly in malaria prevention messaging may also increase their awareness, motivation and accountability.

## INTRODUCTION

Long-lasting insecticidal bednets (LLINs) are the most widely used tool for preventing malaria. Since 2007, the World Health Organization (WHO) has recommended that all people at risk of malaria have access to LLINs, over 2 billion people world-wide^1^. Hundreds of millions of LLINs are distributed every year, typically through universal free distribution programs. These programs have increased ownership of LLINs in African households, which account for 95% of the world’s malaria cases and deaths, from 5% in 2000 to 68% in 2021.

Despite this progress, children in Africa continue to make up more than 75% of the malaria burden in cases and deaths, and access and reported use of bednets among children has stagnated since 2015^2^. In addition, the spread of pyrethroid resistance among malaria vectors has led to the deployment of next generation nets that are significantly more expensive than standard pyrethroid LLINs^3,4^. As the cost of deploying new nets rises, it is increasingly important to understand the factors that affect their use to most cost-efficiently utilize scarce malaria prevention resources.

Despite this progress, children in Africa continue to make up more than 75% of the malaria burden in cases and deaths, and access and reported use of bednets among children has stagnated since 2015^5^. In addition, the spread of pyrethroid resistance among malaria vectors has led to the deployment of next generation nets that are significantly more expensive than standard pyrethroid LLINs^6,7^. As the cost of deploying new nets rises, it is increasingly important to understand the factors that affect their use to most cost-efficiently allocate this prevention tool for malaria control.

Among the many factors that have been associated with individual bednet use, age is the most well-studied^8,9,10^. In general, younger children and adults have better reported bednet use than older, school-aged children. Lower bednet use among school-aged children is significant because this age group may act as a reservoir of infection in the community, due to their partial immunity and lack of symptoms from malaria infection, which can contribute to onward transmission^11,12^. Despite their suboptimal bednet use habits and their importance to malaria transmission in the community, there are only limited studies exploring why school-aged children use their bednets less than other members in the household. The studies that exist typically focus on factors such as access to bednets and sleeping arrangements in the household, highlighting lower prioritization of use among individuals deemed to be less at risk^13,14^. Much less is known about what factors affect use when a bednet is available. The evidence that exists, thus far collected exclusively from adults, suggests that ideational factors such as motivation and habits become increasingly important once access to bednets is adequate^15^. Very little is known about the ideational factors that affect the use of bednets in this age group and we are not aware of any study that has performed in-depth interviews with school-aged children themselves about their bednet use.

This qualitative study was designed to address this gap in our understanding of factors associated with bednet use to prevent malaria. We performed in-depth qualitative interviews about facilitators of and barriers to bednet use with school-aged children whose households were enrolled in a cohort-based study in a high transmission setting in eastern Uganda. This study focuses on themes that arose from those interviews with the goal of developing a deeper understanding of the ways that this crucial group interact with bednets used for the prevention of malaria.

## METHODS

### 2.1 Sampling strategy

We performed a cross-sectional qualitative study with an exploratory research design. Individuals were recruited from households enrolled in a parent cohort study. Details of the cohort have been previously described, but briefly, 80 randomly selected households located in a setting with high transmission of malaria in the Tororo and Busia Districts in Eastern Uganda were enrolled in the cohort and all members of the household were actively followed up every four weeks to monitor for changes in malaria burden^16^. Interviews for this study were performed in a sub-sample of households from the parent study (12 households). These household were enrolled in a sub-study evaluating the use of remote electronic monitors to assess bednet use. The 12 households were purposively sampled from the parent study based on being regular users of bednets. All bednets in the sub-study households were equipped with small watch-sized electronic bednet monitors. Households had access to free clinical care at a study clinic and were visited every 2 weeks for entomological collections and collection of remote bednet monitoring data. The parent study also provided participating households with enough bednets to achieve a ratio of 1 bednet per 2 household inhabitants.

### 2.2. Recruitment and in-depth interview procedures

**Figure 1:**
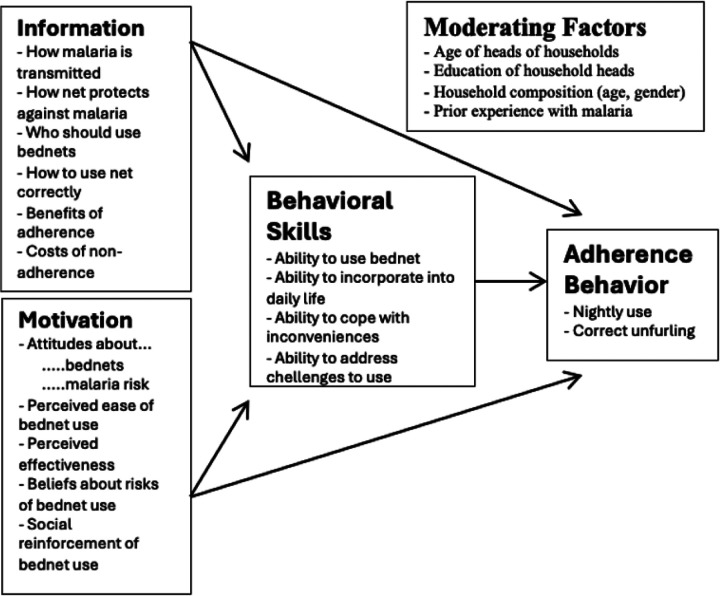
Adapted Information-Motivation-Behavioral model of bednet use

Qualitative data were collected between March and April 2023 through in-depth interviews (IDI). Individuals aged 8 years through 17 years from the target households were identified for participation. Participants were recruited for interviews at monthly scheduled clinic visits that were part of the parent study. The clinic team participated in the screening process, referring participants identified to be from the target households to the social scientist who independently performed the consenting process and interviews. The interviews were conducted immediately after the clinic visit and lasted for about 30 to 45 minutes. The children were interviewed in the presence of an adult caretaker or parent. An interview guide was developed using a behavioral model of bednet use based on an Information-Motivation-Behavioral (IMB) framework (Figure 1)^17^. The interview guide drew upon facilitators and barriers identified in the literature that might affect bednet use, but the structure of the interview guide also allowed for broader exploration of the context around the decision to use bednets. A simplified version of the interview guide was used for children aged 8 through 14 years. In-depth interviews were conducted in English, Dhupadhola, Luganda and Ateso as appropriate. Participants were asked questions such as “What is your understanding of malaria risks?”, “What can you tell me about malaria prevention?”, “How does age and sleeping arrangement affect LLIN use?” “How many rooms do you have at home?”

### 2.3 Reflexivity

The team employed a physician to participate in the screening process. This physician regularly provided clinical care for the participants as part of their enrollment in the parent study. This physician was stationed at Tororo District Hospital and he referred the patients to the social scientist following their scheduled clinic visits. His role in the parent study may have influenced the willingness of participants to participate. The social scientist was a female Ugandan with first-hand experience with the use of bednets and the burden of malaria in Uganda. The social scientist did not participate in any other study activities as part of the longitudinal cohort and did not know the participants. She conducted the IDIs independently with the help of a translator as necessary and the participants were encouraged to freely express themselves and were reminded that their identity would be kept confidential.

### 2.4 Data management and Analysis

Interviews were transcribed verbatim by a team with experience in transcription. Those conducted in local languages were translated into English. The social scientist read through the transcripts to ensure they were a true reflection of the recordings. Data were entered into NVivo software version 14 to aid in organization and data management^18,19^. The transcripts and discussion notes were reviewed for themes and reorganized according to discussion topics. Thematic analysis was carried out guided by the IMB framework. A team-based approach was utilized, with the first author (DES) working in consultation with the senior author (PJK). Data and recurring ideas were coded, and codes were aggregated into themes. This process involved carrying out multiple readings of the field scripts to understand the data and subsequent coding of the data. Coded data extracts were developed into themes presented as sub-topics in this paper. The process was open to identifying new themes from the data, which were then integrated into the pre-existing themes^20^. We applied the consolidated criteria for reporting qualitative research (COREQ) checklist for qualitative studies to the presentation of findings^21^.

## RESULTS

Eighteen children, aged 8 to 17 years, were identified in the 12 households. Ultimately, 5 of the 18 were excluded because of an inability to complete the interview. These 5 participants were excluded due to an inability to understand the questions or an inability to express themselves due to fear or impairment. Therefore, a total of 13 children were interviewed. This included 4 boys and 9 girls, with a mean age of 12.5 years (Standard Deviation: 2.8).

The qualitative data elicited four main themes: barriers to bednet use, facilitators of bednet use, factors that moderate these and potential resolutions for difficulties encountered in consistently using bednets (Table 1).

### THEME 1: BARRIERS TO THE USE OF BEDNETS

Several children reported non-use of the bednets when asked about regular use. This was generally attributed to fatigue, forgetting to use the bednets, parents taking the children’s nets, negative attitudes about net use, inconsistent availability of bednets, parents coming back late and forgetting to check if the children had used the nets and information barriers.

#### Fatigue/forgetting to use

A summary of the findings showed that children who did not use their bednets attributed it to fatigue from school, multiple household chores and those who played a lot during the day. Fatigued children occasionally forgot to use the bednets.

R: *It was last week on Thursday. I over played at school. We played netball at school. On reaching home, I had to do some more work [to help out] at home and at around 8:00pm, I just fell on the bed and slept. Only to wake up in the night and I was not using the net, that’s when I remembered to lower the net*.

(Child 407)

#### Parents don’t give the children their nets

Analysis of the findings showed that children reported that parents had a tendency of keeping the nets that were meant for the children. The parents sometimes kept the nets, used them personally or gave them to their other children in the household.

R. *It’s with my mother, she is the one keeping it. (I. So, who is using your net?) No one is using my net; it is with my mother*.

(Child 406)

#### Negative attitude toward using the net

Children reported that they had negative experiences using the net. Some children reported that they found net use bothersome and those that did were less likely to prioritize their use. Other children reported having negative experiences making them less likely to use the bednet such as physical symptoms like developing a rash on the face which they attributed to bednet use. Others noted having been bitten by mosquitoes despite their use of the bednets and didn’t see the need to continue using the bednet.

R. *When the nets are still new, it causes itching and rash in our faces because of the medicine that has been used for treating the nets*.

(Child 217)

#### Excessive heat

A summary of the findings indicated that excessive heat negatively affected bednet use. Some children reported not wanting anything to cover them or be on top of them when the temperature was high and this negatively affected their use of the nets.

R: *We sleep in the girls’ room and I share a net with my young sister. Sometimes my sister does not want to use a net when it is hot, so she sometimes sleeps aside and I sleep alone. But when it is hot, sometimes we just sleep [under the net but] without covering ourselves*.

(Child 408)

#### Overnight travel

The findings showed that several children reported not using bednets during recent overnight travel. The overnight trips were usually with their parents to funerals or when they went for holidays to other homes during the festive seasons. Children reported that it was hard to carry the nets on these trips and so they were less likely to use bednets when travelling.

#### Inconsistency availability of the bednets

When queried about possible reasons for inconsistency in the use of bednets, children listed washing the net and discovering it had not dried in time for use. Most of the time, the nets were washed by the parents. Other reported reasons were reluctance to hang up the nets, ignorance about the importance of bednet use, and difficulties with changing sleeping spaces leading to a lack of regular sleeping space over which to tie up a net.

#### Inappropriate knowledge

Some of the children appeared ignorant about the importance of bednets in the prevention of malaria. Information barriers that decreased the likelihood of regularly using bednets included myths about the causes of malaria, a lack of information about the importance of preventing malaria and decreased understanding of adequate prevention methods.

### THEME 2: FACILITATORS TO THE USE OF BEDNETS

A summary of the factors that tended to increase the use of bednets included proper knowledge about the causes of malaria and means of prevention, the importance of parental support, adequate supplies of bednets for use and generally positive attitudes about the value of bednets leading to a willingness to use them.

#### Awareness of malaria

Knowledge about causes of malaria, such as being caused by mosquito bites and related to stagnant water, tended to increase the likelihood of children using their nets. Understanding that malaria was preventable by sleeping under mosquito nets and being versed in the benefits of nets in reducing costs spent on treating of malaria among the older children was associated with more bednet use. Children who appeared to be knowledgeable about malaria indicated that the source of this information was from school and their parents.

#### Parental support

The findings showed that children who were supported by their parents by checking on them daily, unfurling their nets and washing their nets early so that they dried in time for use were tended to use nets more than those who didn’t receive similar support.

R: *When I come back then my mother puts it [down] for me, sometimes I don’t even know what time she puts it down but me I just find it*.

(Child 407)

#### Consistency in the use of bednets

Consistency in bednet use was attributed to the fact that participants had more than one net each. Both the government and the study were reported to be the sources of the nets. The adequate supply and ownership of bednets helped in ensuring consistency in use so that when one was washed, they replaced it with another.

R: *The government gave us and even [the study team] also gave us. Actually, we got four from government and also this project gave us four nets, so in total we have eight nets at home*.

(Child 408)

#### Willingness to use the nets

The children indicated that their willingness to use the nets was attributed to the availability of the nets, health workers helping to tie them up and the way that bednets were left hanging during the day so the children wouldn’t forget to unfurl them. Those who thought nets were safe to use were more positive about bednet use. Some children reported the importance of leaving new nets out in the sun before first use to reduce physical reactions/rashes. Additionally, those who found the nets easy to use were more positive about their use than those who found them burdensome. Furthermore, children reported understanding that children and pregnant women should be prioritized during the distribution of nets.

#### The influence of remote bednet monitoring

IDIs among children showed that participation in the remote bednet monitoring study also influenced bednet use. Children reported that the device was small, beautiful and looked like a watch. Some of the participants indicated that they used the nets because they were aware that they were being observed. Since they were being monitored, some children reported more consistently placing the nets in the correct position and replacing them immediately after washing. These behaviors were supported by the health workers who regularly visited the home to manage the monitoring device and leave the net hanging down when they depart.

R: *[The device] looks nice for me. It’s very small, not very big. It’s like a watch. Even if it’s hot, I use the net because this machine will know. I can’t miss. The moment you miss, this machine is going to show there that you have not slept in the net maybe for three days or one day, like that*.

(Child 316)

### THEME 3: MODERATING FACTORS

Moderating factors that affected the use of bednets included age, education, behaviors related to moving around the household at night and structural household factors such as the number of rooms and members in the house.

#### Age

The age of the children had an influence on bednet use. In this study of 8- to 17-year-olds, older children responded more positively to using bednets than the younger children. This appears to be related to the way younger children mainly depended on their parents for support with unfurling the net. Older children also reported being more likely to remember to use nets compared to the younger ones who occasionally got tired after a long day of play or household chores. One of the older children noted regarding bednet use while sharing a bed with a younger child:

R: *If she doesn’t want to sleep under the net, I leave her out. I will not allow to get malaria because of her*.

(Child 596)

#### Education

In general, both the uneducated and educated groups of children seemed to have understood the importance of using bednets. Those who were better educated appreciated the benefits of prevention more and reported more reminders to help them remember. This seemed to be attributed to the information they gained from school and their parents about the importance of malaria prevention.

#### Nighty movements

Several children reported occasions when they exited bednets during the night to use the toilet. Multiple of these narratives indicated that they routinely returned to find that they had left the net open, allowing mosquitoes to enter the net. This left them vulnerable to disturbance and bites after these interruptions in use occurred:

R: *Most times, when I go to the toilet in the night, I try to cover, but when I come back I hear the sound of mosquitoes inside the net*.

(Child 219)

#### Household factors

Household factors appear to have an influence on bednet use. Participants from homes with more rooms seemed more likely to use bednets consistently than those with few rooms. Participants with fewer rooms indicated that the use of bednets was made more challenging because they often had to sleep in non-standard sleeping spaces, such as the sitting room, where hanging up permanent nets was more difficult. Limited sleeping spaces relative to the number of household inhabitants was therefore correlated with participants being less likely to regularly use bednets. Those with more rooms and with permanent sleeping positions indicated that they found it easy to use the bednets. In addition, sharing beds with other individuals, primarily with other children, sometimes causes difficulties when there is a discrepancy in wanting to use the net.

### THEME 4: RESOLUTIONS

Children were engaged in a discussion about ways to resolve the barriers to bednet use that they reported during their interviews. Potential resolutions were most often discussed by the children in the context of parental support as a key first step in enacting changes. A summary of some of these potential resolutions included children identifying the value of additional nets so that one is available for their use and to avoid interruptions after washing. Children also noted the importance of allowing sufficient time in their days to unfurl the net and for making this a habit that was guided by their typical daily routine. The children indicated the importance of putting the nets down early and some noted that leaving the nets down all of the time, instead of folding them up during the day, might be a way to help avoid forgetting. The older children identified the need to wash nets and hang them to dry early to ensure they are dried and ready to be put back before the night.

## DISCUSSION

This study explored factors that affect bednet use among school-aged children living in a high transmission setting in Uganda by interviewing them directly about their knowledge, practices and beliefs. In this study of 13 children, aged 8 to 17 years, multiple facilitators of bednet use and barriers to their regular use were identified. Some findings have been reported previously among adults, such as heat being a barrier to use and concerns about new nets causing skin irritation. Nonetheless, this study focusing exclusively on children provides multiple new perspectives on circumstances around bednet use. Some of the most interesting findings for understanding how children experience bednet use, and the prevention of malaria more generally, include the key role of parents, how the daily activities and routines of children affect use, and challenges specific to children related to sharing sleeping spaces with others, limited availability of nets and lack of dedicated sleeping spaces. These findings can help inform public health campaigns and policy makers seeking to improve the uptake of bednet use in malaria endemic settings.

Children within the 8 to 17 age range are in a developmental stage marked by increased independence and a growing sense of agency and have been found to be the least likely to use bednets. Studies conducted in Africa support our findings, showing that school-aged children are generally less likely to use bednets. Studies in Uganda have shown that bednet use was consistently lowest among school-aged children (5 to 17 years)^22,23^. This may be related to findings that children over 5 years of age are perceived to be at less risk for malaria because they tend to have fewer clinical episodes due to partial immunity^24,25,26^. Nonetheless, very few studies have explored bednet use practices by children over 5 years of age and we are not aware of any studies that have assessed this with direct interviews with children.

An important finding in this study is the influence of parents as gatekeepers for bednet use. Mothers of younger children were more likely to be responsible for unfurling and washing the nets compared to older children who take on the responsibilities themselves. The attitude of parents and caretakers towards bednets therefore directly affects use of bednets of school-aged children, particularly among the younger ages. It seems possible that parents with a positive attitude toward bednet use might be more likely to prioritize and ensure bednet use among their children. This is born out in the literature, with caretakers who perceived malaria as a less serious disease being less likely to ensure bednet use for their children compared to those who perceived malaria as deadly with significant economic implications^27^. In many cultures, the care and distribution of bednets in a home, ensuring they are washed and used are considered the role of women in the home^28^. These societal expectations of mothers regarding bednet use for children create the possibility that they are key gatekeepers to their use. When mothers get overwhelmed or forget to unfurl the bednets for their children, those children appear to be at increased risk of malaria as a result.

School-aged children as a group are typically characterized by high energy levels and a strong leaning towards physical activity. It is plausible that they get tired after a long day of play and that this may contribute to their forgetfulness regarding bednet use. The findings of our study on the influence of play align with studies by which were conducted in Tanzania^29,30^. In addition, children are involved in routine domestic activities which may affect their malaria exposure, such as fetching water, washing kitchen utensils and cooking, and walking to and from school. Watching television and studying late at night have also been reported as activities that keep school-aged children outdoors in the early evening and night hours, thus increasing their exposure to malaria.

Access to bednets is another important theme that arose from our study. Research has shown that this age group is often perceived as being lower risk and therefore has lower priority in the household allocation of bednets for the prevention of malaria. This age group bears an underappreciated burden of malaria and there are few targeted interventions towards this age category^31^. The permanence of sleeping spaces arose as a key factor affecting bednet use in our study. Children who had to share beds and bednets and those who did not have permanent sleeping positions reported more barriers to bednet use compared to those with permanent dedicated sleeping spaces. This aligns with findings in other work^24^.

In addition, there were several barriers and facilitators of bednet use we elicited from our school-aged child participants that confirm what is known about bednet use in general. Some of the these include logistical matters such as bednets not having dried after being washed and bednets being torn. These findings are consistent with other findings from Uganda where the most common reason cited for non-usage of bednets the previous night was that the net was not hung^32^. Temperature also has an effect on bednet use in Uganda, with prior reports that during excessive heat and in the dry season bednets are rarely used^33^. In addition, some people in Uganda use bednets only during the rainy season and put it away during the dry season^34^. Overnight travel of children with their parents for social events is another factor found to affect consistent bednet use in Uganda and this also was revealed in our study^35,36^

Among the limitations of this study, we relied only on the experiences of the children, which may have left under-explored the perceptions of their parents and other household members. Furthermore, these interviews were retrospective, potentially introducing recall bias into responses. Children were also interviewed in the presence of their parents, which may have affected their willingness to report poor bednet use and how candid they could be about matters affecting their bednet use, particularly as they related to those parents. Finally, the households available for these interviews were purposively sampled for their regular bednet use. It is possible that there would be differences if we interviewed a random sampling of children from the community.

Overall, while these school-aged children recognized the importance of preventing malaria and generally held a favorable view towards the use of bednets, maintaining consistent usage proved to be difficult. Individual factors such as forgetfulness and fatigue, induced by expected levels of activities for this age group (i.e. play and chores), negatively impacted the regular use of bednets. Interventions for improving the development of bednet use habits among school-aged children, as they transition into independent decision-makers of their own health, can improve net use culture in Uganda now and in the future. Parents were also revealed as key gatekeepers to bednet use, particularly among younger school-aged children. It is essential for parents and caregivers to actively support children by monitoring their use of bednets during the night. Finally, access to bednets and regular sleeping spaces were identified as determinants of bednet use in this age group. These findings can help public health programs and policy makers develop interventions to address these barriers to bednet use in this age group.

## Supplementary Files

This is a list of supplementary files associated with this preprint. Click to download.

- InterviewguidebednetuseVersfinal.docx

## Figures and Tables

**Table 1.** Summary of qualitative results.

Themes	Sub-themes
*Barriers hindering bednet use*	Inadequate Knowledge/Misinformation
	Fatigue
	Negative attitude towards bednet use
	Dependence on parents to unfurl the nets
	Excessive heat Sharing beds
	Inappropriate knowledge
	Inconsistency in the availability of nets
	Overnight travel
*Facilitators improving bednet use*	Malaria knowledge Presence of the electronic monitoring device
	Willingness to use the bednet
	Influence of the smart nets
	Parental support by mothers
*Moderating factors*	Age
	Education level
	Nightly movements
	Structural factors
*Resolutions*	Have available extra nets
	Make time for drawing the net
	Provide nets for those who lack
	Put the net down early
	Willing to wash the net early and hang it to dry so it is available for use

## Data Availability

Source data in the form of recorded voices have not been shared because they could break anonymity. The IDI guide and data analyzed for this manuscript is available from the corresponding author upon request.

## Abbreviations

IDI: In-depth interviews
IMB: Information-Motivation-Behavioral
IRB: Institutional Review Board
LLIN: Long-lasting insecticidal bednets
WHO: World Health Organization
